# High STRN Expression Promotes HCC Invasion and Migration but Not Cell Proliferation or Apoptosis through Facilitating Epithelial-Mesenchymal Transition

**DOI:** 10.1155/2020/6152925

**Published:** 2020-03-23

**Authors:** Qian-yu Du, Jing-hao Yao, Yong-chun Zhou, Ling-jie Xu, Fu-you Zhao, Yan Yang

**Affiliations:** ^1^Department of Medical Oncology, The First Affiliated Hospital of Bengbu Medical College, Bengbu 233004, China; ^2^Department of Radiation Oncology, The First Affiliated Hospital of Bengbu Medical College, Bengbu 233004, China

## Abstract

A *STRN-ALK* fusion protein has been recently identified as a potential therapeutic target in multiple cancers; however, the role of STRN alone in regulating the biological function of hepatocellular carcinoma (HCC) remains unclear. In this study, we firstly detected an overexpression of STRN in HCC tissues compared to that in adjacent nontumour (ANT) tissues through IHC analysis, and the expression level of this protein was positively correlated with lymph node metastasis and TNM stage. *In vitro*, high expression of STRN was also confirmed in different HCC cell lines, and regulation of STRN expression in Huh7 cells did not significantly affect tumour cell proliferation or apoptosis but was positively correlated with tumour cell invasion and migration capacities. Moreover, both the knockdown and overexpression of STRN in Huh7 cells can lead to cell morphological changes that are accompanied with an alteration of epithelial-mesenchymal transition (EMT) molecular markers E-cadherin and Vimentin. Finally, STRN was further proved to be negatively related to E-cadherin expression but positively related to Vimentin expression in human HCC tissue samples. Taken together, STRN is upregulated in HCC and acts as a tumour promoter regulating cell invasion and migration through facilitating the EMT process.

## 1. Introduction

Liver cancer is the fourth most lethal cancer worldwide, and the main pathological type is hepatocellular carcinoma (HCC) [1]. China has the largest number of HCC cases and deaths worldwide, accounting for more than 50% of the total, and HCC has unique characteristics [2]. The early symptoms of HCC are hidden, causing most patients to be in the middle and late stages of the disease at the time of diagnosis, thereby losing the opportunity for surgery. Moreover, even with radical resection, more than 50% of HCC patients may experience tumour relapse and metastasis [3]. Although great improvement has been achieved in HCC diagnosis and therapy, the prognosis of HCC is still very poor, and this inferior prognosis is related to HCC's strong proliferative, antiapoptotic, and invasive abilities. Numerous studies suggest that epithelial-mesenchymal transition (EMT) is closely related to the invasion and migration of HCC [4–6] and acts as a potential therapeutic target in HCC treatment [7]. EMT refers to the process of cell remodelling characterized by the loss of polarized organization and acquisition of migratory and invasive capabilities by epithelial cells upon multiple physiological and pathological stimuli. The decrease in E-cadherin expression and an increase in Vimentin expression are the most important molecular features of EMT [8]. However, the concrete regulatory factors and networks initiating EMT are not fully elucidated.

STRN (striatin) is a protein that is encoded by the STRN gene in humans and is an important member of the striatin family, which includes STRN, STRN3 (SG2NA), and STRN4 (zinedin). Members of the striatin family feature multiple protein-binding domains, such as a caveolin-binding motif, a coiled-coil structure, a calmodulin-binding site, and a WD-repeat domain. These domains can mediate the dimerization of members of the striatin family and their interaction with a series of proteins [9]. The *STRN-ALK* fusion gene, a new driver gene, has been found in multiple cancers, including thyroid cancer [10], non-small-cell lung cancer (NSCLC) [11], colorectal cancer [12], and renal carcinoma [13]. Due to the activity involved in the regulation of multiple protein kinases that play a role in tumour progression, striatin family proteins themselves perform as a link to tumours. Indeed, STRN4 is highly expressed in a variety of tumour cells, and STRN4 knockdown suppresses the proliferation, invasion, and metastasis of cancer cells both *in vitro* and *in vivo* and increases the sensitivity of pancreatic cancer cells to gemcitabine *in vitro* [14]. However, the biological function of STRN in HCC is undefined. There are no relevant studies regarding STRN expression in HCC. Therefore, in the present study, we first revealed the expression and clinical significance of STRN in human HCC tissues and further investigated its effect on HCC cell biological behaviour, including cell proliferation, apoptosis, invasion, and migration, and finally explored the possible molecular mechanisms. Our results indicate a possible role of STRN in HCC invasion and migration through facilitating the EMT process.

## 2. Materials and Methods

### 2.1. HCC Patients and Tissue Samples

Forty-five samples of HCC after surgical resection were collected from the First Affiliated Hospital of Bengbu Medical College, China, between July 2014 and June 2016. The patients received no preoperative treatment, and complete clinical data were obtained. The pathological grade was defined by the Edmondson and Steiner classification. The baseline characteristics of the HCC patients are listed in Table 1. In addition, adjacent nontumour (ANT) tissues, which were diagnosed as normal by pathological methods, were obtained at a distance of 5 cm or more from the tumour tissue in patients with HCC. Approval was obtained from the Medical Ethics Committee of Bengbu Medical College (Bengbu, China), and written informed consent was obtained from the patients or their immediate family members.

### 2.2. Immunohistochemistry (IHC)

IHC was carried out on 4 *μ*m thick, formalin-fixed, paraffin-embedded sections according to the two-step protocol that we described previously [15, 16]. Anti-STRN (1 : 50, Proteintech), anti-E-cadherin (1 : 50, Abcam), and anti-Vimentin (1 : 100, Abcam) primary antibodies were used for immunohistochemical analysis. Specific procedures were strictly performed according to the manufacturer's instructions. The intensity of positive signals was scored as follows: 0, negative (no staining); 1, weak (light yellow); 2, moderate (yellowish-brown); and 3, strong (brown). The extent of positivity was scored based on the percentage of positive cells: 0, <5%; 1, 5%~25%; 2, 26%~50%; 3, 51%~75%; and 4, >75%. The staining index (SI) was obtained as the final score by multiplying the above scores, yielding a score ranging from 0 to 12. When staining is heterogeneous, each score is independent, and the results are aggregated. Then, the median SI value of 6 was selected as the cutoff, and samples with SI ≥ 6 and SI < 6 were assigned to the high and low expression groups, respectively.

### 2.3. Cell Line and Cell Culture

The human normal liver cell line LO2 was purchased from KeyGEN Biotech (Nanjing, China). The human HCC cell lines Huh7, SMMC-7721, and HepG2 were provided by the Cell Bank of the Chinese Academy of Sciences (Shanghai, China). Cells were cultured in DMEM supplemented with 10% foetal bovine serum, 100 U/ml streptomycin, and 100 mg/ml penicillin at 37°C in a humidified atmosphere containing 5% CO_2_.

### 2.4. Gene Overexpression and Silencing

The knockdown of human STRN was performed by transient transfection of siRNA. We set the negative control (NC) and STRN siRNA as the control and experimental groups, respectively. The target sequences of NC and STRN siRNA are presented in Table 2. All the plasmids were delivered into the cells by Lipofectamine™ 2000 (Invitrogen). The knockdown efficiency was measured by Western blot.

For overexpression purposes, the full-length cDNA of human STRN (Gene ID: 6801) was cloned into the pcDNA3.1, which was delivered into cells with Lipofectamine™ 2000 (Invitrogen). The cells transfected with solitary pcDNA3.1 were set as the control group. The expression of STRN was examined by Western blot.

### 2.5. Quantificational Real-Time Polymerase Chain Reaction (qRT-PCR)

Total RNA was extracted using TRIzol (Invitrogen) reagent, and the cDNA was synthesized using Reverse Transcription System (Promega) according to the manufacturer's instructions. cDNA was amplified following the SYBR Premix Ex Taq™ kit (TaKaRa) protocol, and the appropriate primers are listed in Table 3. Real-time PCR was initiated at 95°C for 10 min followed by 40 cycles of 95°C for 30 sec and 60°C for 30 sec. Amplification signals for samples were normalized to the internal control gene (GAPDH). Fold change of gene expression was calculated by the 2^−*ΔΔ*Ct^ method.

### 2.6. Western Blot

Cells were lysed in RIPA lysis buffer with protease inhibitor cocktail, and protein concentration was determined using the BCA Protein Assay Kit (Thermo Fisher). 20 *μ*g protein and adequate loading buffer were added per well, denatured in a 95°C water bath, and fractionated by SDS-PAGE. Subsequent to electrophoresis, the proteins were electronically transferred onto a polyvinylidene difluoride filter membrane, blocked with 5% skim milk for 1 h at room temperature, and coincubated overnight with the appropriate anti-human primary antibody on a shaker at 4°C. The primary antibodies and dilutions used for Western blot were as follows: anti-STRN (1 : 500; Abcam), anti-E-cadherin (1 : 1,000; Abcam), anti-Vimentin (1 : 1,000; Abcam), and anti-*β*-actin (1 : 500; Santa Cruz Biotechnology). Protein expression levels were normalized to those of the internal control (*β*-actin).

### 2.7. Immunofluorescence Assay

Immunofluorescence assay was performed according to the standard protocols that we described previously [15, 16]. Samples were blocked with 2% BSA in PBS and probed with primary antibodies (anti-STRN, 1 : 50; anti-E-cadherin, 1 : 200; and anti-Vimentin, 1 : 200) overnight at 4°C. Samples were then incubated with FITC-conjugated secondary antibodies (1 : 200, diluted in 2% BSA) for 1 h in the dark at room temperature. The cells were examined and imaged under a fluorescence microscope (Olympus). Images were then quantified and relativized against DAPI signal, and data were obtained using ImageJ 1.48 software.

### 2.8. MTT Assay

Cell proliferation was assessed by an MTT assay according to our previous studies [17, 18] with some modifications. Parental and transfected Huh7 cells were seeded at a density of 7 × 10^3^ cells/well into 96-well plates for the indicated time periods. Cells were then incubated with 15 *μ*l MTT (5 mg/ml) for 4 h, and the medium was replaced with 150 *μ*l 100% dimethylsulfoxide (DMSO) in each well. Finally, the absorbance at 490 nm was determined using an ELx800 UV universal microplate reader (BioTek).

### 2.9. Annexin V/PI Staining

Cell apoptosis was measured by an Annexin V-FITC and propidium iodide (PI) (BD Biosciences Clontech, USA) labelling technique and flow cytometric analyses, and the staining was carried out according to the method recommended by the manufacturer, as described in our previous report [18].

### 2.10. Transwell Invasion Assay

Invasion assay was performed using a transwell system (8 *μ*m pore size; Millipore). Cell invasion capability was measured with 24-well chambers precoated with Matrigel (BD Biosciences) on the polycarbonate membrane. 3 × 10^4^ cells suspended in 200 *μ*l of serum-free medium were seeded in the upper compartment of the transwell chambers, and 1 ml of complete medium was added to the lower compartment. After an incubation period of 48 h, the cells remaining in the upper chamber were scraped using a sterile cotton-tipped applicator, whereas the cells that have invaded through the Matrigel were fixed and stained with 0.1% crystal violet for 30 min. The inserts were then imaged and counted using a light microscope.

### 2.11. Wound Healing Assay

Cells were cultured to form a confluent monolayer in 6-well plates. After starvation, the cell monolayer was scratched with a 200 *μ*l sterile tip to mimic a wound, and incubation was continued for 48 h. Images were taken at 0 and 48 h after scarring under an inverted microscope (Olympus). The wound area was quantified using ImageJ 1.48 software.

### 2.12. Statistical Analysis

All statistical analyses were performed using the GraphPad Prism version 8.0 statistical software (San Diego, CA, USA). Numerical data are the means ± SD and were compared by an unpaired Student's *t*-test. Differences between two groups in immunohistochemical analysis were evaluated by the chi-squared test or Fisher's exact test as appropriate. Spearman correlation was used to analyse the association of the expression of STRN and EMT markers. Differences with *P* < 0.05 were considered significant.

## 3. Results

### 3.1. Expression and Clinical Significance of STRN in HCC

**Figure 1:**
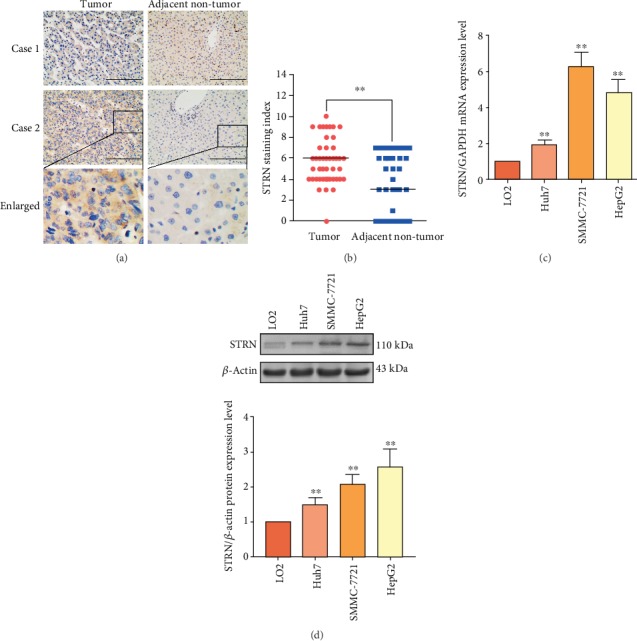
Expression of STRN in HCC tissues and cells. (a) Staining of STRN was performed by immunohistochemistry, and representative images from two cases are shown. Scale bars, 200 *μ*m. (b) The staining index (SI) of STRN was significantly higher in HCC tissues than in ANT samples. (c) The mRNA expression level of STRN in the human normal liver cell line LO2 and the HCC cell lines Huh7, SMMC-7721, and HepG2 was measured by qRT-PCR. (d) The protein expression level of STRN in the human normal liver cell line LO2 and the HCC cell lines Huh7, SMMC-7721, and HepG2 was determined by Western blot analysis. ^∗∗^*P* < 0.01*vs.* Tumor (b) and LO2 (c, d).

STRN was mainly expressed in the cytoplasm, and its protein expression level in HCC tissues was significantly higher than that in ANT tissues (Figure 1(a)). Among the 45 specimens, only 1 (1/45, 2.2%) HCC specimen showed negative STRN expression, while the others showed either low or moderate expression of STRN. Among the ANT tissues, there were 15 (15/45, 33.3%) specimens showing negative STRN expression, while the remaining specimens mostly exhibited low levels of the indicator. Statistical analysis showed that the SI of STRN in HCC tissues was significantly higher than that in ANT tissues (Figure 1(b)). STRN expression was not significantly associated with patient age, sex, tumour size, tumour histologic grade, cirrhosis or hepatitis background, intrahepatic vascular embolism, aminotransferase level, alpha-fetoprotein (AFP) level, C-reactive protein level, serum albumin level, or prothrombin time (all *P* > 0.05), but was positively correlated with lymph node metastasis and TNM stage (both *P* < 0.05) (Table 1). In addition, STRN expression at both mRNA (Figure 1(c)) and protein (Figure 1(d)) levels was markedly upregulated in the three HCC cell lines (SMMC-7721, Huh7, and HepG2) compared to that in the LO2 cell line *in vitro*. Notably, due to the only expected size (110 kDa) but no larger STRN band was observed, we believe that the protein detected in Western blot corresponds to STRN and not to any of its fusion proteins. Thus, we histologically and cytologically confirmed an increased STRN expression during hepatocarcinogenesis.

### 3.2. Downregulation of STRN Had No Significant Effect on HCC Cell Proliferation and Apoptosis but Decreased the Invasion and Migration Capacities of HCC Cells

**Figure 2:**
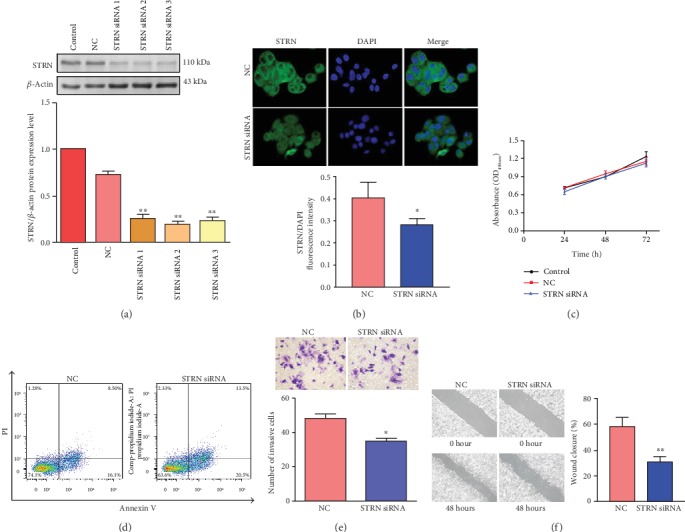
Effect of STRN depletion on the proliferation, apoptosis, invasion, and migration capacities of Huh7 cells. (a) Western blot was conducted to detect the inhibitory effect of STRN siRNA in Huh7 cells. (b) Inhibition of STRN in Huh7 cells was confirmed by immunofluorescence assay (original magnification, ×200). (c) The Huh7 cell proliferation potential was measured by MTT assay upon STRN downregulation via siRNA. (d) Cell apoptosis was assessed using flow cytometric analysis in STRN-downregulated Huh7 cells. (e) The invasive ability of Huh7 cells was suppressed by STRN knockdown, as assessed by the transwell invasion assay. Representative bright-field microscopy images are shown (original magnification, ×200). (f) The migratory capacity of Huh7 cells was inhibited by STRN knockdown, as assessed by the wound healing assay (original magnification, ×100). ^∗^*P* < 0.05 and ^∗∗^*P* < 0.01*vs.* NC. Abbreviation: NC: negative control.

To clarify the role of STRN in HCC progression, we first aimed to silence specific STRN expression via siRNA transfection in the HCC cell line Huh7. Western blot analysis showed that the expression of STRN was significantly downregulated, with siRNA2 exhibiting the greatest inhibitory effect (Figure 2(a)). Immunofluorescence assays also confirmed the successful establishment of the STRN-knockdown Huh7 cell model (Figure 2(b)). Subsequently, downregulation of STRN induced no effect on cell proliferation and apoptosis, as shown by the MTT assay (Figure 2(c)) and flow cytometric analysis (Figure 2(d)). However, a decrease in tumour cell invasion and migration capacities could be observed by transwell invasion (Figure 2(e)) and wound healing assays (Figure 2(f)), upon STRN inhibition.

### 3.3. Upregulation of STRN Had No Significant Effect on HCC Cell Proliferation and Apoptosis but Increased the Invasion and Migration Capacities of HCC Cells

**Figure 3:**
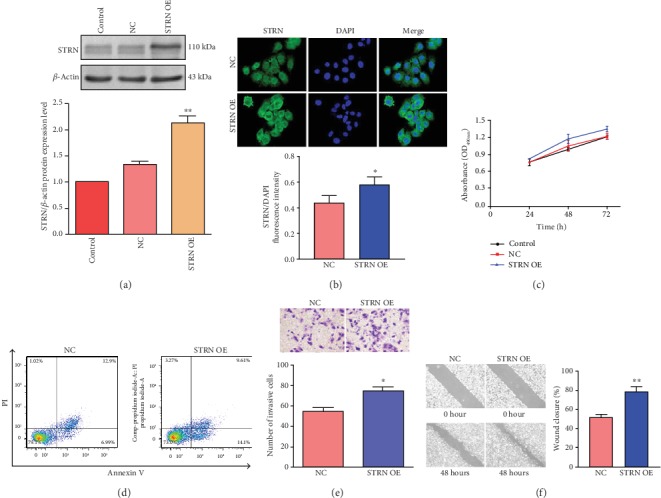
Effect of STRN overexpression on the proliferation, apoptosis, invasion, and migration capacities of Huh7 cells. (a) Western blot analysis was performed to confirm the overexpression of STRN in Huh7 cells following transfection with pcDNA3.1/hSTRN. (b) Enhanced STRN expression in Huh7 cells transfected with STRN cDNA was confirmed by immunofluorescence assay (original magnification, ×200). (c) Cell proliferation potential was measured by MTT assay in Huh7 cells transfected with STRN cDNA. (d) Cell apoptosis was assessed using flow cytometric analysis in STRN-upregulated Huh7 cells. (e) Transwell invasion assay was conducted to measure the invasive capacity of Huh7 cells overexpressing STRN, and representative bright-field microscopy images are shown (original magnification, ×200). (f) Wound healing assay was performed to investigate the migratory potential of STRN-overexpressed Huh7 cells (original magnification, ×100). ^∗^*P* < 0.05 and ^∗∗^*P* < 0.01*vs.* NC. Abbreviations: NC: negative control; OE: overexpression.

To achieve our goals, we further transfected Huh7 cells with STRN cDNA to upregulate STRN expression. Western blot analysis showed that the expression of STRN was significantly upregulated in Huh7 cells following transfection with STRN cDNA (Figure 3(a)). Immunofluorescence also confirmed the successful establishment of the STRN-overexpressed Huh7 cell model (Figure 3(b)). Similarly, no effect of STRN overexpression on cell proliferation and apoptosis was shown by the MTT assay (Figure 3(c)) and flow cytometric analysis (Figure 3(d)). However, an increase in the invasion and migration capacities of Huh7 cells overexpressing STRN was found in transwell invasion (Figure 3(e)) and wound healing assays (Figure 3(f)). These results showed a positive role of STRN in HCC cell progression by enhancing the tumour invasion and migration capacities but not the cell proliferation or apoptosis potential.

### 3.4. STRN Affected HCC Cell Invasion and Migration Abilities via Its Effect on EMT

**Figure 4:**
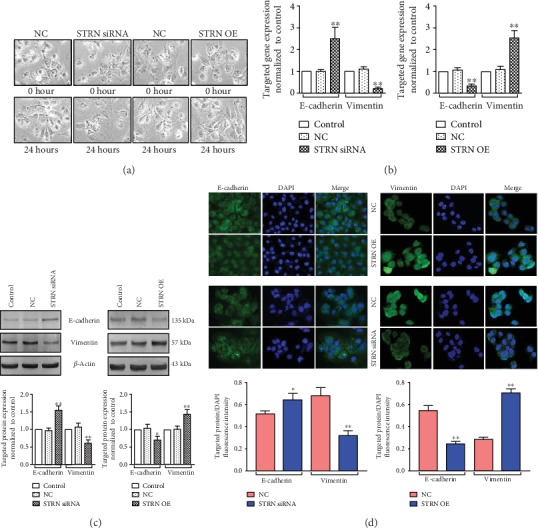
Modulation of STRN expression in Huh7 cells influences the EMT or MET process *in vitro*. (a) Morphological changes of Huh7 cells with STRN downregulation and overexpression were observed by microscopy, and representative images are shown (original magnification, ×200). (b) qRT-PCR assay was performed to measure the mRNA level of the epithelial marker E-cadherin and the mesenchymal marker Vimentin in Huh7 cells with dual-directional regulation of STRN expression. (c) Effect of STRN on the expression of EMT-related proteins. E-cadherin and Vimentin were examined in Huh7 cells with STRN downregulation and overexpression by Western blot analysis. (d) Immunofluorescence assay was performed to assess the expression of markers of epithelial and mesenchymal phenotypes (original magnification, ×200). ^∗^*P* < 0.05 and ^∗∗^*P* < 0.01*vs.* either control or NC (b, c); ^∗^*P* < 0.05 and ^∗∗^*P* < 0.01*vs.* NC (d). Abbreviations: NC: negative control; OE: overexpression.

Since the migration and invasion of tumour cells are closely related to EMT, we further investigated whether STRN has the potential to affect EMT in HCC cells. During the process of dual-directional regulation of STRN in Huh7 cells, cell morphological changes exhibiting an epithelioid and cobblestone appearance with little pseudopodia were observed by microscopy upon STRN downregulation. In contrast, STRN-overexpressed Huh7 cells showed increased formation of pseudopodia, leading to an elongated, irregular fibroblastoid morphology (Figure 4(a)). Moreover, qRT-PCR, Western blot, and immunofluorescence assays confirmed an increase in the expression of the epithelial marker E-cadherin but a decrease in the expression of the mesenchymal marker Vimentin upon STRN suppression. However, an opposite result was observed in the expression of EMT markers by STRN upregulation (Figures 4(b)–4(d)). These results suggest that STRN might affect HCC cell invasion and migration capacities through EMT regulation.

### 3.5. Correlation between STRN Expression and EMT Marker Expression in Human HCC Tissues

**Figure 5:**
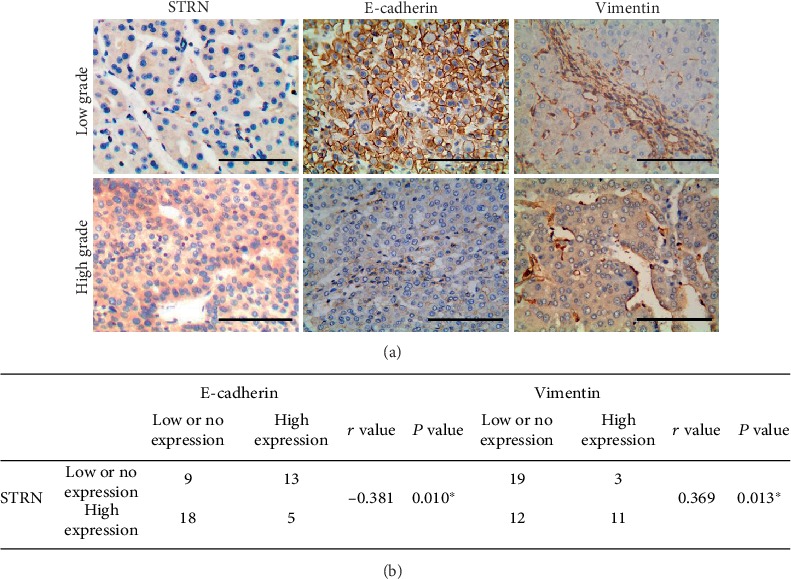
Correlation of STRN expression with E-cadherin and Vimentin expression in human HCC tissues. (a) Representative staining of STRN, E-cadherin, and Vimentin in HCC tissues by immunohistochemical analysis. Images from a low-grade HCC, which exhibits low STRN expression, positive E-cadherin membranous staining, and negative Vimentin expression, and images from a high-grade HCC, which exhibits high STRN expression, reduced E-cadherin membranous staining, and positive Vimentin expression, are shown. Scale bars, 100 *μ*m. (b) STRN expression was negatively correlated with E-cadherin expression but positively correlated with Vimentin expression in HCC tissues, as analysed by Spearman correlation.^∗^*P* < 0.05.

As mentioned earlier, STRN was mainly located in the cytoplasm of cancer cells and was highly expressed in human HCC tissues (Figures 1(a), 1(b), and 5(a)), with a positive rate of high expression of 51.1% (23/45) (Figure 5(b)). E-cadherin was abundantly expressed in the cell membrane in ANT tissues (data not shown) but showed predominantly low expression in HCC tissues (Figure 5(a)). The rate of high E-cadherin expression in HCC tissues was only 40.0% (18/45), and the loss of membrane expression in HCC tissues with low differentiation was the most significant (Figures 5(a) and 5(b)). Vimentin was generally not expressed in normal hepatocytes but was highly expressed in the cytoplasm in HCC tissues, and the positive rate of high Vimentin expression in HCC tissues was 31.1% (14/45) (Figures 5(a) and 5(b)). Spearman correlation analysis showed that STRN expression was negatively correlated with E-cadherin expression and positively correlated with Vimentin expression (Figure 5(b)). Thus, these results confirmed the regulatory relationships among STRN, E-cadherin, and Vimentin during hepatocarcinogenesis.

## 4. Discussion

Gene fusion in cancer is important clinically because it is often the primary driver of oncogenesis and renders the host tumour highly sensitive to targeted therapy. The rare *STRN-ALK* fusion has been found in several different cancer types, including papillary thyroid cancer [10], NSCLC [11], colorectal cancer [12], and renal carcinoma [13]. Functionally, gene fusion involving STRN leads to constitutive activation of ALK kinase via dimerization mediated by the 5′ coiled-coil domain in the gene [10], while investigation of the role of the gene itself in tumour progression was neglected in most studies. To date, the biological function of STRN, an important member of the striatin family, in HCC is undefined. In the current study, we first analysed STRN expression in HCC tissues and its clinical relevance and investigated the potential biological function of STRN in HCC cells. Our results suggest that STRN is upregulated in HCC tissues and cells and acts as a tumour promoter regulating cell invasion and migration through facilitating the EMT process.

STRN, a multidomain scaffolding protein, has been demonstrated by numerous studies to be indicated in several biological processes [19, 20]. The relationship between STRN and tumours has attracted researchers' attentions in recent years. For example, STRN3 was reported to activate the Akt signalling pathway to enhance the survival of cancer cells [21]. Silencing of the STRN4 protein in many tumour cells can inhibit their proliferation, invasion, and migration [14]. In addition to its association with multiple kinases, STRN can function as a regulatory subunit of protein phosphatase 2A (PP2A) [22]. PP2A represents an abundant class of structurally complex serine/threonine phosphatases found in all eukaryotes that regulates a host of cellular processes via phosphorylation and dephosphorylation [23]. Evidence indicates that PP2A displays proapoptotic functions in human cancer cells [24] and favours the suppression of malignant transformation [25]. However, other studies have demonstrated that PP2A also counteracts apoptotic cell death, by showing that inhibition of PP2A increases progression of cell cycle in the presence of cytotoxic chemotherapeutic drugs [26], and also enhances radiation-induced mitotic catastrophe and tumour growth delay in glioblastoma [27], thereby facilitating the effectiveness of various cancer therapies. We hypothesized that the seemingly contradictory functions may be related to the distinct striatin-associated PP2As, as proteins of the striatin family can differ in their cell type-specific gene expression patterns and biological functions [28]. In the present study, we revealed that STRN may promote HCC invasion and migration but does not significantly affect cell proliferation or apoptosis, strengthening the support for the important but complicated role of STRN in the development of malignant tumours.

Accumulating evidence has shown that EMT plays a crucial role in HCC invasion and metastasis [4–6]; EMT is characterized by the loss of epithelial cell polarity and cell-cell contacts by downregulation of E-cadherin expression and upregulation of the expression of mesenchymal markers such as Vimentin [8]. Our previous investigation revealed that a partial EMT process was often activated in HCC tissues [15, 16], supporting the hypothesis that EMT is partially responsible for HCC progression through mediating cell migration, invasion, and metastasis. In the present study, overexpression of STRN enhanced the migration and invasion capacities of Huh7 cells and induced the EMT phenotype. Silencing STRN produced the opposite results. We confirmed the relationship between STRN and the EMT markers E-cadherin and Vimentin in human HCC tissues. The potential of STRN in regulating the invasion and migration abilities of HCC cells was also confirmed histologically; the expression of STRN was positively correlated with unfavourable clinicopathological parameters such as lymph node metastasis and advanced TNM stage in HCC patients by analysis of clinicopathological characteristics. Notably, STRN protein expression was not correlated with tumour size or histological classification, consistent with the observation that the regulation of STRN expression *in vitro* did not significantly affect the proliferation or apoptosis of Huh7 cells.

## 5. Conclusions

In summary, the present investigation revealed that STRN was aberrantly overexpressed in HCC tissues and positively correlated with tumour lymph node metastasis and clinical stage. The increased expression of STRN was found to contribute to EMT in HCC cells, which could partially explain the role of STRN in promoting HCC invasion and migration but not cell proliferation or apoptosis *in vitro*. Taken together, our findings indicate that STRN alone is involved in HCC progression by inducing EMT.

## Figures and Tables

**Table 1:** Relationship between STRN expression and clinicopathological parameters of 45 HCC samples.

Variable	*n*	STRN		*χ* ^2^ value	*P* value
		Low	High		
Age (years)					
<60	31	11	20	0.000	1.000
≥60	14	5	9		
Gender					
Male	39	14	25	0.000	1.000
Female	6	2	4		
Tumour size (cm)					
≤5	37	14	23	0.079	0.779
>5	8	2	6		
Edmondson type					
I–II	31	14	17	2.778	0.096
III–IV	14	2	12		
TNM stage					
I–II	24	12	12	4.683	0.030^∗^
III–IV	21	4	17		
Hepatopathy background					
Present	42	14	28	0.293	0.589
Absent	3	2	1		
Lymph node metastasis					
Negative	26	13	13	5.607	0.018^∗^
Positive	19	3	16		
Intrahepatic vascular embolism					
Present	4	0	4	1.018	0.313
Absent	41	16	25		
Aminotransferase					
Higher	20	4	16	3.802	0.051
Normal	25	12	13		
Alpha-fetoprotein (AFP)					
Higher	30	10	20	0.194	0.660
Normal	15	6	9		
C-reactive protein					
Higher	19	4	15	3.019	0.082
Normal	26	12	14		
Serum albumin					
≥35	35	15	20	2.371	0.124
<35	10	1	9		
Prothrombin time					
Higher	20	5	15	1.751	0.186
Normal	25	11	14		

^∗^ *P* < 0.05; *χ*^2^ test.

**Table 2:** siRNA sequences for human STRN.

Gene	Sequence	
	Sense (5′-3′)	Antisense (5′-3′)
STRN siRNA1	GCACAGAGGCUGAAGUUAATT	UUAACUUCAGCCUCUGUGCTT
STRN siRNA2	GCAUUGACAUUUCCUCCUUTT	AAGGAGGAAAUGUCAAUGCTT
STRN siRNA3	CCAAGAAUUCACAGCUCAUTT	AUGAGCUGUGAAUUCUUGGTT
Control	UUCUCCGAACGUGUCACGUTT	ACGUGACACGUUCGGAGAATT

**Table 3:** The primers used for qRT-PCR analysis.

Gene	Sequence		Product size (bps)
	Sense (5′-3′)	Antisense (5′-3′)	
STRN	ACGAGTGCGAGCTTTGTTG	TGCTGTCTCTTTAACTTCAGCC	109
E-cadherin	GAAGTGTCCGAGGACTTTGG	CAGTGTCTCTCCAAATCCGATA	109
Vimentin	TGTCCAAATCGATGTGGATGTTTC	TTGTACCATTCTTCTGCCTCCTG	117
GAPDH	TGACTTCAACAGCGACACCCA	CACCCTGTTGCTGTAGCCAAA	121

## Data Availability

The data that support the findings of this study are available from the corresponding author upon reasonable request.
